# Immunogenicity of three-dose COVID-19 vaccines in people living with multiple sclerosis

**DOI:** 10.1136/bmjno-2025-001210

**Published:** 2025-12-16

**Authors:** Marianne J Shawe-Taylor, David Greenwood, Anna He, Agnieszka Hobbs, Giulia Dowgier, Rebecca Penn, Theo Sanderson, Phoebe Stevenson-Leggett, James Bazire, Ruth Harvey, Dimitrios Champsas, Suraiya Sharmin, Anuriti Aojula, Alessia Bianchi, Sarmad Al-Araji, Yael Hacohen, Charmaine Yam, Suraya Mohamud, Ronja Christensen, Marcello Moccia, Crick COVID Serology Pipeline, Ashley S Fowler, Legacy Investigators, Rupert CL Beale, Vincenzo Libri, George Kassiotis, Steve Gamblin, Nicola Lewis, Bryan Williams, Charles Swanton, Sonia Gandhi, David Bauer, Mary Wu, Edward Carr, Emma Wall, Olga Ciccarelli

**Affiliations:** 1 The Francis Crick Institute, London, UK; 2 National Institute for Health Research (NIHR) University College London Hospitals (UCLH) Biomedical Research Centre and NIHR UCLH Clinical Research Facility, UCLH, London, UK; 3 National Hospital for Neurology and Neurosurgery, London, UK; 4 Department of Neuroscience, Monash University, Melbourne, Victoria, Australia; 5 COVID Surveillance Unit, The Francis Crick Institute, London, UK; 6 Worldwide Influenza Centre, The Francis Crick Institute, London, UK; 7 National Institute for Health Research (NIHR) University College London Hospitals (UCLH) Biomedical Research Centre and NIHR UCLH Clinical Research Facility, University College London Hospitals NHS Foundation Trust, London, UK; 8 Department of Infectious Disease, St Mary’s Hospital, London, UK; 9 UCL, London, UK; 10 University College London Cancer Institute, London, UK; 11 Genotype-to-Phenotype 2 Consortium, The Francis Crick Institute, London, UK; 12 Neurology and Neuroscience Biomedical Research Centre, UCL, London, UK

**Keywords:** multiple sclerosis, COVID-19

## Abstract

**Introduction:**

People with multiple sclerosis (pwMS) receiving B-cell depleting disease-modifying therapy (BCD-DMT) are vulnerable to severe COVID-19. Data on vaccine immunogenicity in this patient group are incomplete. In the context of the rapid evolution of SARS-CoV-2 2020–22, we compared vaccine responses in pwMS and healthy vaccinated adults (HVA) after three doses of messenger RNA vaccine encoding Ancestral SARS-CoV-2 Spike.

**Methods:**

In this prospective observational cohort study, we collected serum from 226 pwMS prevaccine and postvaccine and quantified neutralising antibody titres (nAbT) in a high-throughput live virus assay against SARS-CoV-2 Ancestral, Alpha, Delta, Omicron BA.1, BA.2 and BA.5. We compared nAbT in pwMS and HVA, matched by age, sex, vaccine type, number of doses and time since exposure, using Wilcoxon signed-rank and χ^2^ tests. We further investigated nAbT vaccine response in pwMS on BCD-DMTs or non-depleting DMTs.

**Results:**

Prior to third vaccination, nAbTs against nearly all variants tested were significantly lower (p<0.05) in pwMS taking BCD therapy than those in HVA or B-cell replete pwMS, and were not significantly boosted following vaccination. In contrast, B-cell replete pwMS versus HVAs exhibited equivalent prevaccination nAbTs against all variants, which were comparably boosted against most variants following vaccination. Consequently, differences in nAbTs against all variants tested were further magnified between B-cell replete and B-cell depleted pwMS post-third vaccination. Across the entire cohort, there were no COVID-19 hospitalisations or deaths. Notably, sera collected prior to the pandemic from pwMS demonstrated pre-existing, pan-coronavirus neutralising activity against seasonal HCoV-OC43 and SARS-CoV-2 variants.

**Conclusions:**

PwMS taking BCD therapy have limited antibody boosting following repeated COVID-19 vaccination. However, the absence of severe outcomes in pwMS, despite reduced immunogenicity, suggests a lower threshold for effective protection than previously reported. These findings support more nuanced risk stratification in clinical policy.

**WHAT IS ALREADY KNOWN ON THIS TOPIC**

- Before this study, people with multiple sclerosis (pwMS) were considered vulnerable to severe COVID-19 due to neurological disability and long-term use of disease-modifying therapies (DMTs), especially B-cell depleting therapies (BCDT).
- Early reports suggested pwMS on BCDT had a reduced antibody response to vaccines and higher risk of hospitalisation or death from COVID-19 compared with pwMS on other therapies; however, pwMS were excluded from the original vaccine efficacy trials, and existing data on vaccine-induced neutralising antibody titres (nAbTs) were limited to small, early pandemic studies that did not capture responses to later SARS-CoV-2 variants.

**WHAT THIS STUDY ADDS**

- This study demonstrates that pwMS on non-BCDT mount vaccine responses comparable to healthy adults, while pwMS on BCDT have persistently lower nAbTs following a third COVID-19 vaccine dose.
- Importantly, reduced antibody titres in pwMS on BCDT did not translate into increased risk of COVID-19 hospitalisation, suggesting partial protection was maintained.
- The study also provides novel evidence of cross-reactive neutralising activity in pwMS sera collected before COVID-19 emerged, implying a potential role for pre-existing coronavirus immunity.

**HOW THIS STUDY MIGHT AFFECT RESEARCH, PRACTICE OR POLICY**

- These findings support tailoring vaccination strategies for pwMS, with particular attention to timing and duration of BCDT when planning vaccine schedules.
- The results challenge the assumption that all pwMS remain highly vulnerable to severe COVID-19, suggesting that the ‘vulnerable’ designation could be cautiously revised for those not on BCDT.
- Future research should focus on long-term antibody waning, the contribution of cross-reactive immunity and identifying protective immune correlates to guide booster and treatment recommendations in pwMS.

## Introduction

 People with multiple sclerosis (pwMS) have been considered vulnerable to severe COVID-19 caused by SARS-CoV-2, due to both neurological disability and exposure to immunomodulatory treatments.^1^ Central nervous system reactive T-lymphocytes and B-lymphocytes are implicated in the pathogenesis of multiple sclerosis (MS).^2^ Disease-modifying therapies (DMTs) for MS target immune mechanisms. Anti-CD20 DMTs deplete B cells to interrupt B-cell-T-cell interactions and suppress acute inflammatory disease activity to reduce both new radiographic lesions and clinical relapses.^3^ B-cell depleting therapies (BCDT) are used for approximately 200 000 pwMS worldwide.^4^ Other DMTs target T cells, cytokines or inflammatory pathways.^5^ PwMS often receive DMTs for years, uninterrupted, to sustain remission.^6^ Anti-CD20 drugs impair effective vaccine-mediated immunity by reducing antibodies generation, consequently reducing the effectiveness of tetanus and influenza vaccines, among others.^7^ PwMS were therefore included on the UK’s ‘Shielded Patient List’ of 2020, advising them to stay at home and minimise contact with others and were prioritised by the UK Joint Committee on Vaccination and Immunisation (JCVI) in the first COVID-19 vaccination programme. PwMS continue to be offered updated vaccines^8^ and treatments like antivirals,^9^ but little data support this ongoing recommendation.

The risk of severe COVID-19 varies widely among pwMS, determined by: DMT exposure, age, disability, comorbidities and socioeconomic status.^10^ PwMS treated with BCDT have a higher risk of mortality and hospitalisation from COVID-19 compared with other DMTs.^1^ This risk is lower than people with haematological or rheumatological diseases on BCDT.^11^ Before vaccination, the estimated COVID-19 mortality risk in pwMS ranged from analogous to the general population to up to 65% higher risk.^12^ Since the delivery of COVID-19 vaccines in 2021, mortality and morbidity from COVID-19 have dramatically declined in the global population, including pwMS.^13^ However, pwMS were excluded from the original vaccine trials, and clinical vaccine-efficacy and immunogenicity data remain limited to small early pandemic studies.^14 15^ Data relevant to later variants or vaccines are scarce. As COVID-19 continues to evolve to escape both infection and vaccine-mediated immunity, ongoing assessments of vaccine efficacy are required for pwMS, to accurately risk stratify and inform protection for this vulnerable patient group.

To test the hypothesis that pwMS have a blunted antibody response to vaccination, we characterised longitudinal neutralising antibody titres (nAbT) against sequential SARS-CoV-2 variants in a unique cohort of pwMS at University College London Hospitals, under infection surveillance 2020–2022, stratified by DMT. We focused on the response to the third dose of the three-dose prime-boost COVID-19 vaccine regime to compare optimal vaccine responses between individuals receiving differential DMT and matched healthy vaccinated adults (HVAs) in the University College London Hospitals (UCLH)-Crick Legacy cohort. We then tested for associations between nAbT, duration of BCDT and subsequent infection risk.

## Methods

### Design

Prospective observational cohort study.

### Participants and setting

#### PwMS—the UCL COVMS study

We recruited participants with a diagnosis of MS into this single-centre prospective study hosted by the National Hospital for Neurology and Neurosurgery (NHNN), the UK’s largest centre of neurology and neurosurgery. Eligible participants were aged >16 years and able to comply with the study protocol. We collected metadata from electronic health records, including demographic information, COVID-19 infection episodes and recorded DMT use since diagnosis. We obtained COVID-19 vaccine information from the National Immunisation and Vaccination System. Serum samples were collected during regular clinic visits. Some COVMS participants were enrolled in other NHNN studies prior to COVMS. Samples taken from these were included in the COVMS analysis.

#### Healthy vaccinated adults: the UCLH-Crick Legacy study

The Legacy study (NCT04750356) is a prospective observational cohort, established by the UCLH National Institute for Health Research Biomedical Research Centre and the Francis Crick Institute, sponsored by UCLH. Participants were adults who had undergone COVID-19 testing or vaccination and who were employed by UCLH, the Francis Crick Institute, Central and North West London Foundation Trust or Ealing and Northwick Park Hospitals. Participants underwent regular serological and virological surveillance. Extensive descriptions of the cohort can be found in prior reports.^16 17^ We collected serum samples during study visits and approximately 2–3 weeks after COVID-19 vaccination or infection. Legacy participants were matched 1:1 to the COVMS cohort by: age, sex, vaccine type, number of vaccine doses, number of COVID-19 exposures (vaccine or infection) and time since last exposure.

### Assays

#### SARS-CoV-2 live virus neutralisation assay

We quantified nAbT against six SARS-CoV-2 variants (Ancestral, Alpha, Delta, Omicron BA.1, BA.2 and BA.5) using our high-throughput microneutralisation assay at the COVID Surveillance Unit at the Francis Crick Institute, as previously described.^18^ We also tested the assay against HCoV-OC43, a seasonal coronavirus that circulated before COVID-19, to test for potentially cross-reactive antibodies.^19^ The assay has been calibrated to the WHO International Standard for anti-SARS-CoV-2 immunoglobulin.^20^

#### Antinucleocapsid IgG detection

We measured antinucleocapsid IgG (anti-N IgG) using the Elecsys Anti-SARS-COV-2 assay (Roche; 09203095190) run on a Cobas e411 analyser (Roche) in accordance with the manufacturer’s instructions. Serum was used for this immunoassay and results reported as reactive (positive) or non-reactive (negative).

### Exposures

We compared pwMS exposed to BCDT with those not receiving BCDT (non-DT). For analysis of duration of BCDT, we used time between the administration of the first dose of treatment and sampling date.

### Covariates

Covariates included in all analyses were: age at time of serum sample and sex. For analyses using time points after any COVID-19 exposure, we included the following parameters: (1) type of vaccine(s) given, (2) time of vaccine(s) relative to serum sample, (3) time of infection(s) relative to serum sample and (4) total number of exposures (vaccines and infections) prior to serum sample.

### Outcomes

To analyse longitudinal serological profiles, we defined the outcomes as: nAbTs against seasonal coronaviruses and SARS-CoV-2 variants across the pandemic (including prior to infection or vaccine exposure). For analysis of vaccine response, the outcome was nAbTs pre-third and post-third vaccine exposure. COVID-19 infection was the outcome event for infection risk. We censored events if participants received another COVID-19 vaccine dose or began treatment with BCDT before infection.

### Definitions

Exposure to B-cell depletion: treatment with alemtuzumab, ocrelizumab or ofatumumab at the time of sampling.

COVID-19 infection episode: detectable anti-N antibody, positive lateral flow or PCR swab, attendance to a COVID medicines delivery unit (CMDU) with a positive swab or symptoms in keeping with COVID-19.

Pre-third vaccine dose samples: samples taken before their third dose and >12 weeks after their second dose, with no recent COVID-19 infection episode (<12 weeks prior).

Post-third vaccine dose samples: samples taken <12 weeks after the third dose, and before the fourth dose, with no recent COVID-19 infection episode (<12 weeks prior).

Sample taken prior to SARS-CoV-2 antigen exposure: sample collected before the first dose of vaccine; no evidence of previous COVID-19 infection episode; sample negativity for antinucleocapsid (N) and anti-Spike (S) IgG.

### Data analysis, statistics and availability

We collected Legacy study data and COVMS data using the Research Electronic Data Capture, hosted at University College London. We imported data from both studies into R V.4.3.1 prior to analysis as previously described. We manipulated, analysed and visualised the data using the *chronogram* and *tidyverse* R packages including *dplyr* and *ggplot2*. We produced summary descriptions of the cohorts using *gtsummary* and reported continuous data as the median value and IQR or the first and third quartiles (Q1; Q3). For statistical tests, we used the *rstatix* R package.

We performed an analysis of nAbTs in serum as previously described without alterations using unpaired two-tailed Wilcoxon signed-rank tests. We estimated fold-change (FC) between groups with a 95% CI with the *boot* R package using 5000 bootstrap resamples. If median antibody titres for either group fell outside of the quantifiable range of the assay (inhibitory concentration 50% (IC_50_) <40 or >2560), we assessed the number of sera that did (or did not) have quantifiable titres for statistical significance using χ^2^ tests. For any given comparison, the method used is indicated on the relevant panel.

For plotting and analysis, we used winsorising: IC_50_ values above the quantitative limit of detection of the assay (>2560) were recoded as 5120; IC_50_ values below the quantitative limit of the assay (<40) but within the qualitative range were recoded as 10; data below the qualitative range, indicating no observed response, were recorded as 5. These adjustments do not impact any statistical parameters assessed in the analysis (ie, they fall beyond the median and IQR). Moreover, we refrained from conducting analyses based on the absolute values of the points; instead, we employed rank-based analyses.

We conducted time-to-event analysis using the *survival* and *survminer* R packages. We assessed the cumulative incidence of infection by taking the complement of the Kaplan-Meier estimator and applying a log-rank test.

We conducted tobit regression for censored data using the *VGAM* R package. We regressed nAbTs on age, sex, anti-N IgG, time-relative to third dose of COVID-19 vaccine and time-relative to start of B-cell depletion. We censored IC_50_ values below or above the quantitative limit of detection of the assay at 40 and 2560, respectively. Statistical tests were conducted with likelihood ratio tests comparing the full model and as a nested reduced model with and without the term for duration of BCDT, measured in weeks.

## Results

### Participants

We enrolled 228 pwMS in the COVMS study between 28 January 2020 and 25 April 2022, all of whom had sequential serum samples available. The median age was 39 (IQR 32–48), 139 (62%) were female and 89 (39%) were male (table 1). Two hundred and fourteen (94%) participants had received at least three doses of a COVID-19 vaccine. One hundred and five participants (46%) received BNT162b2 (Pfizer-BioNTech) for their first and second doses, and 177 (83%) for their third dose. One participant (0.5%) received a third dose of bivalent BNT162b2+BA1 (Pfizer-BioNTech). AZD1222 (Oxford-AstraZeneca) and Spikevax (Moderna) were given as the first and second doses in 117 (52%) and four (1.8%) participants, respectively. Three (1.4%) participants received AZD1222 as their third dose and 33 (15%) received Spikevax. At the time of sampling, 135 (59%) participants were on BCDT, 79 (35%) were on non-B-cell depleting DMTs and 14 (6%) were not receiving any DMT (table 1). Of the 228 pwMS included, we were able to match 174 (76%) participants’ HVA samples from the UCLH-Crick Legacy study.

**Table 1:** Baseline characteristics of the entire COVMS cohort

Characteristic	PwMS not taking BCDT, n=93*	PwMS taking BCDT, n=135*
Sex		
Female	59 (63%)	80 (59%)
Male	34 (37%)	55 (41%)
Median age (years) (IQR)	39 (32−49)	39 (31−47)
Number of COVID-19 infections		
0	71 (76%)	102 (76%)
1	14 (15%)	15 (11%)
2	6 (6.5%)	11 (8.1%)
3	2 (2.2%)	5 (3.7%)
4	0 (0%)	2 (1.5%)
Number of attendances at a CMDU		
0	89 (96%)	119 (88%)
1	4 (4.3%)	14 (10%)
2	0 (0%)	2 (1.5%)
EDSS score		
≤4	11 (55%)	47 (73%)
4–7	8 (40%)	17 (27%)
>7	1 (5.0%)	0 (0%)
Unknown	73	71
Hospitalised	0 (0%)	0 (0%)
DMT course at time of sampling		
Ocrelizumab	0 (0%)	126 (93%)
Ofatumumab	0 (0%)	1 (0.7%)
Other B-cell depleting	0 (0%)	8 (5.9%)
Natalizumab	65 (70%)	0 (0%)
Other non-B-cell depleting	14 (15%)	0 (0%)
No DMT	14 (15%)	0 (0%)
First dose		
AZD1222	50 (55%)	67 (50%)
BNT162b2	38 (42%)	67 (50%)
mRNA-1273	3 (3.3%)	1 (0.7%)
Unknown	2	0
Second dose		
AZD1222	50 (55%)	67 (50%)
BNT162b2	38 (42%)	67 (50%)
mRNA-1273	3 (3.3%)	1 (0.7%)
Unknown	2	0
Third dose		
AZD1222	0 (0%)	3 (2.3%)
BNT162b2	71 (85%)	106 (82%)
BNT162b2+BA1	1 (1.2%)	0 (0%)
mRNA-1273	12 (14%)	21 (16%)
Unknown	9	5

* N (%); median (25%−75%).

BCDT, B-cell depleting therapies; DMT, disease-modifying therapy; EDSS, Expanded Disability Status Scale; mRNA, messenger RNA; PwMS, people with multiple sclerosis.

### Trends in variant-specific neutralising antibody titres 2020−2023

**Figure 1:**
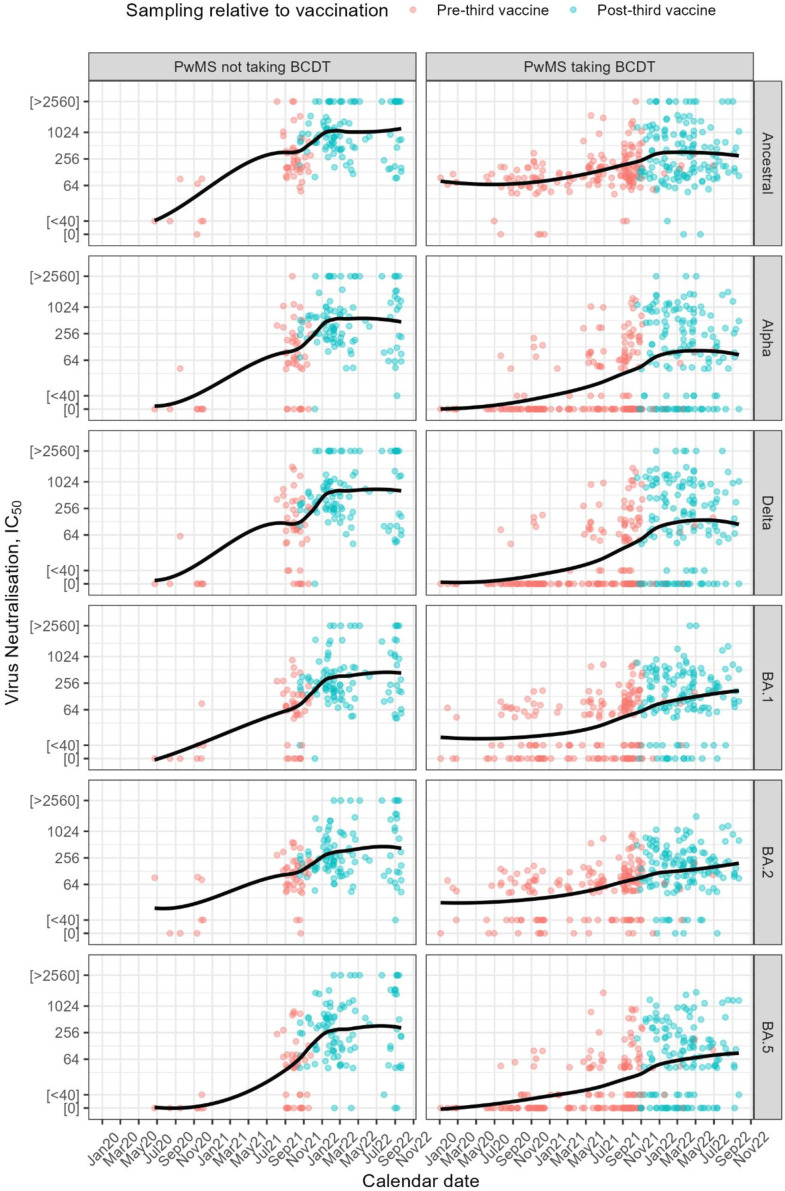
Neutralising antibody titres increased in people with multiple sclerosis (pwMS) over the course of the pandemic. Serum neutralisation titres (IC_50_) against the indicated SARS-CoV-2 variant are plotted for pwMS, taking B-cell depleting therapy or not (pwMS taking B-cell depleting therapies (BCDT) and pwMS not taking BCDT, respectively) between 2020 and 2023. Sera drawn before (‘pre-third vaccine’) and after (‘post-third vaccine’) the third vaccination are plotted in red and blue, respectively.

We first considered the trajectories of nAbTs between 2020 and 2023 in pwMS. nAbT against SARS-CoV-2 variants (Ancestral, Alpha, Delta, Omicron BA.1, BA.2 and BA.5) increased over the course of the pandemic in pwMS, with trajectories differing by B-cell depletion status (figure 1). In line with data on sustained nAbT after a prime/boost course of three doses of COVID-19 vaccines,^21^ we found nAbTs increased primarily after 21 September 2021, when the National Health Service (NHS) third dose vaccine began, in both groups, but nAbT rose more slowly in those on BCDT than those on non-DT, and titres in the BCDT were overall lower. To minimise confounding by time and determine optimal nAbT after a prime-boost three-dose vaccine regime, we therefore subsequently focused on the period of nAbT increment and analysed dose three responses.

### Third dose boosting of nAbT against all variants in pwMS

**Figure 2:**
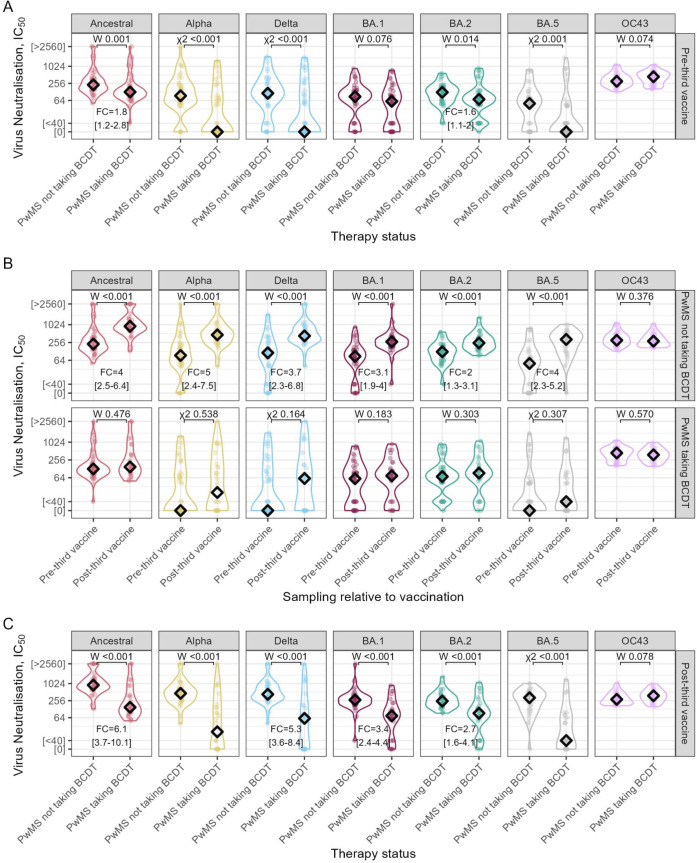
People with multiple sclerosis (pwMS) not taking B-cell depleting therapy (BCDT) showed enhanced ability to neutralise SARS-CoV-2 variants after a third dose compared with their depleted counterparts. Serum neutralisation titres (IC_50_) against SARS-CoV-2 variants and HCoV-OC43 in samples collected before a third dose of BNT162b2 messenger RNA (mRNA) vaccine (pre-third vaccine) or after (post-third vaccine) for pwMS, taking BCDT or not (pwMS taking BCDT and pwMS not taking BCDT, respectively). (A) IC_50_ for pwMS pre-third vaccine by therapy status. (B) IC_50_ for pwMS by sampling relative to vaccination. (C) IC_50_ for pwMS post-third vaccine by therapy status. In each panel, p values were determined using Wilcoxon signed-rank tests (W), or, if a group median value was below the limit of detection, a χ^2^ test was used (see ‘Methods’ section for further details). Median fold-change (FC) between groups is calculated with a 95% CI.

We first tested whether BCDT blunted nAbT responses to third dose vaccination (table 2). Before the third dose, pwMS taking BCDT had significantly lower nAbTs than pwMS taking non-DT, across all SARS-CoV-2 variants except Omicron BA.1 (p=0.08 in BA.1, p<0.05 in all other variants, figure 2A). PwMS on non-DT showed a significant increase in nAbTs after the third dose (p<0.001 across all variants, figure 2B). In contrast, pwMS on BCDT showed no significant postdose boost (p>0.05 across all variants, figure 2B). PwMS taking non-DT had significantly higher nAbTs post-third dose than pwMS taking BCDT, across all variants (p<0.001 across all variants, figure 2C). The nAbTs boosting in pwMS on non-DT ranged between an FC of 2.0 (95% CI 1.3 to 3.1, for Omicron BA.2) to 5.0 (95% CI 2.4 to 7.5, for Alpha). There was no significant difference in HCoV-OC43 nAbTs predose or postdose between BCDT and non-DT groups (p>0.05, figure 2A and C), nor did COVID-19 vaccination increment nAbTs against HCoV-OC43 in pwMS (p>0.05, figure 2B).

**Table 2:** Baseline characteristics of COVMS participants included in the third dose analysis

Characteristic	PwMS not taking BCDT			PwMS taking BCDT		
	Paired samples taken prevaccine and postvaccine, n=14*	Samples taken prevaccine only, n=27*	Samples taken postvaccine only, n=21*	Paired samples taken prevaccine and postvaccine, n=4*	Samples taken prevaccine only, n=46*	Samples taken postvaccine only, n=24*
Sex						
Female	7 (50%)	21 (78%)	13 (62%)	3 (75%)	32 (70%)	7 (29%)
Male	7 (50%)	6 (22%)	8 (38%)	1 (25%)	14 (30%)	17 (71%)
Median age (years) (IQR)	42 (37–55)	37 (32–50)	44 (36–49)	33 (30–37)	42 (34–47)	46 (33–53)
Hospitalised	0 (0%)	0 (0%)	0 (0%)	0 (0%)	0 (0%)	0 (0%)
DMT course at time of sampling						
Natalizumab	9 (64%)	20 (74%)	16 (76%)			
Other non-B-cell depleting	2 (14%)	3 (11%)	2 (9.5%)			
No DMT	3 (21%)	4 (15%)	3 (14%)			
Ocrelizumab				4 (100%)	46 (100%)	24 (100%)
Other B-cell depleting				0 (0%)	0 (0%)	0 (0%)
First dose						
AZD1222	6 (43%)	17 (63%)	8 (38%)	4 (100%)	21 (46%)	12 (50%)
BNT162b2	7 (50%)	9 (33%)	13 (62%)	0 (0%)	25 (54%)	12 (50%)
mRNA-1273	1 (7.1%)	1 (3.7%)	0 (0%)			
Second dose						
AZD1222	6 (43%)	17 (63%)	8 (38%)	4 (100%)	21 (46%)	12 (50%)
BNT162b2	7 (50%)	9 (33%)	13 (62%)	0 (0%)	25 (54%)	12 (50%)
mRNA-1273	1 (7.1%)	1 (3.7%)	0 (0%)			
Third dose						
BNT162b2	14 (100%)	27 (100%)	21 (100%)	4 (100%)	46 (100%)	24 (100%)

* N (%); median (25%–75%).

BCDT, B-cell depleting therapies; DMT, disease-modifying therapy; mRNA, messenger RNA; PwMS, people with multiple sclerosis.

**Figure 3:**
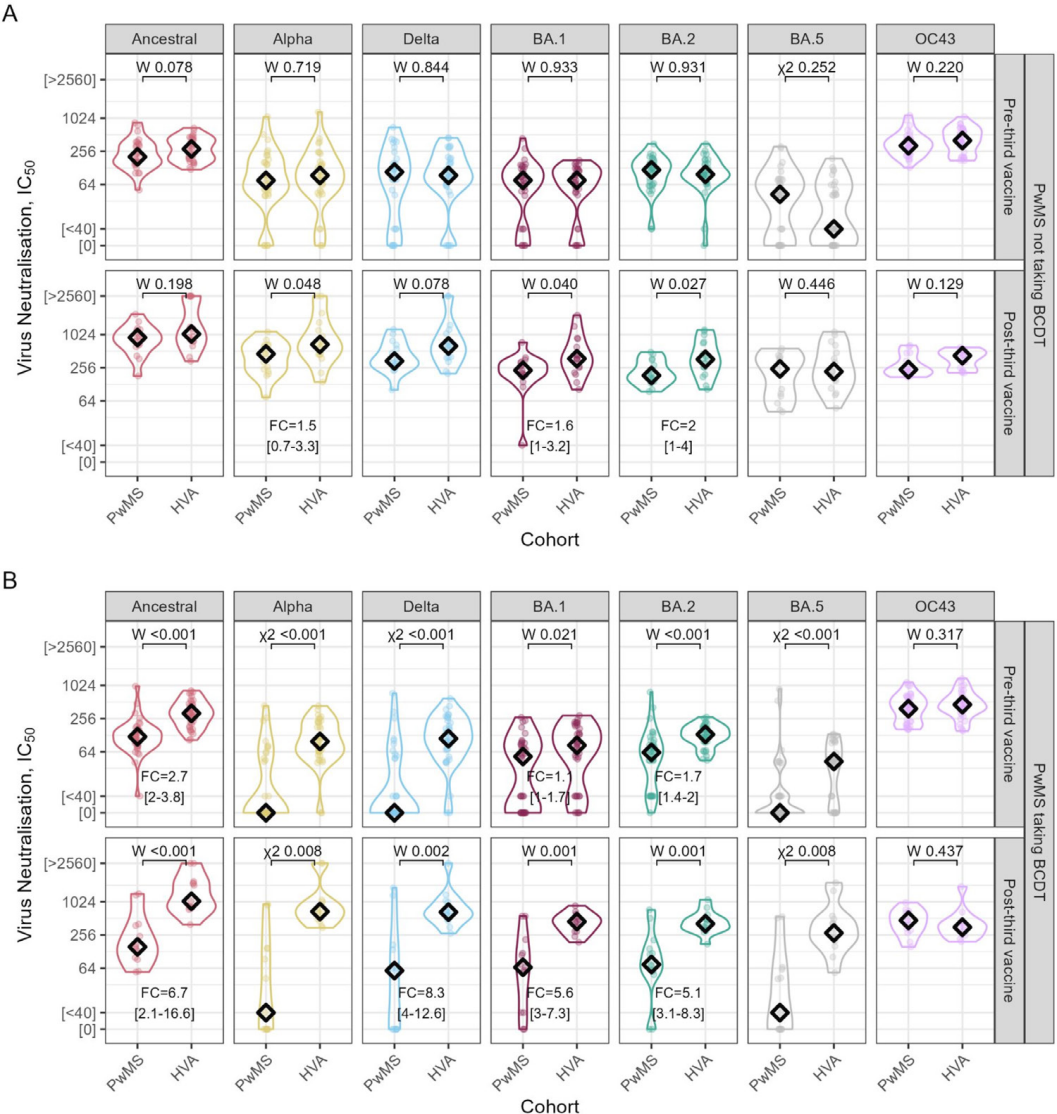
After a third dose, healthy vaccinated adults (HVA) exhibit neutralisation titres comparable to that of the people with multiple sclerosis (pwMS) not receiving B-cell depleting therapy (BCDT). Serum neutralisation titres (IC_50_) against SARS-CoV-2 variants and HCoV-OC43 in samples collected before a third dose of BNT162b2 messenger RNA (mRNA) vaccine (pre-third vaccine) or after (post-third vaccine) for HVA and pwMS, taking BCDT or not (pwMS taking BCDT and pwMS not taking BCDT, respectively). (A) IC_50_ pre-third and post-third vaccine for pwMS not taking BCDT and HVA. (B) IC_50_ pre-third and post-third vaccine for pwMS taking BCDT and HVA. In each panel, p values were determined using Wilcoxon signed-rank tests (W), or, if a group median value was below the limit of detection, a χ^2^ test was used (see ‘Methods’ section for further details). Median fold-change (FC) between groups is calculated with a 95% CI.

Having confirmed the muted dose three response in pwMS taking BCDT, we then compared responses in pwMS with HVA from the UCLH-Crick Legacy study (online supplemental table 1). NAbTs in the pwMS cohorts were contrasted with HVA sera at matched time points (table 3). Before the third vaccine dose, we observed no significant differences in nAbTs in the pwMS on non-DT compared with HVAs (p>0.05, figure 3A). In contrast, pwMS on BCDT had significantly lower pre-third dose nAbTs against all SARS-CoV-2 variants (p<0.05 across all variants, figure 3B). We found a similar increase in nAbTs post-third dose between the pwMS on non-DT pwMS and HVAs against all variants, except for Omicron BA.1, where nAbTs post-dose were 1.6-fold higher (95% CI 1.0 to 3.2, p=0.04) in HVAs than in pwMS on non-DT (figure 3A). In contrast, pwMS on BCDT had significantly lower post-dose nAbTs against all variants, compared with both pwMS on non-DT and HVAs (p<0.05 across all variants, figures2A  3B). Regardless of B-cell depletion, pwMS had comparable nAbTs against HCoV-OC43 to HVAs (p>0.05, figure 3A,B).

**Table 3:** Sampling date summary of third vaccine doses

Cohort	Samples taken predose	Samples taken postdose
COVMS Median sampling date (IQR)	29 September 2021 (8 September 2021–13 October 2021)	14 December 2021 (20 November 2021–14 January 2022)
Legacy Median sampling date (IQR)	21 July 2021 (30 June 2021–24 September 2021)	24 November 2021 (24 October 2021–20 December 2021)

### COVID-19 infection risk in vaccinated pwMS

**Figure 4:**
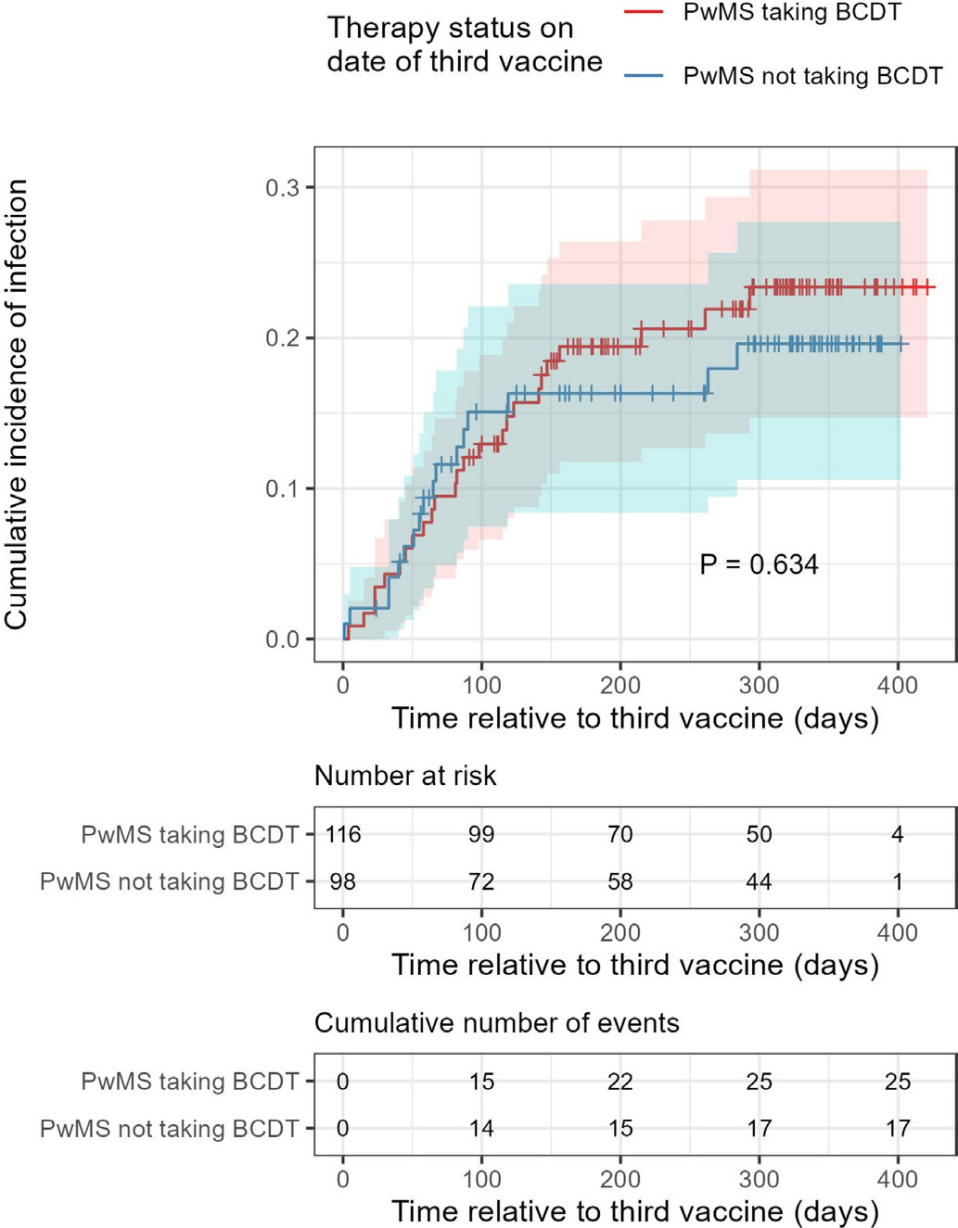
Reduced neutralisation titres after B-cell depleting therapy (BCDT) did not translate to a significant increased probability of SARS-CoV-2 infection. Kaplan-Meier estimates of the probability of SARS-CoV-2 infection after a third dose of BNT162b2 mRNA vaccine for people with multiple sclerosis (pwMS), taking BCDT or not (pwMS not taking BCDT and pwMS taking BCDT, respectively). Statistical significance was assessed using a log-rank test.

During the study period, pwMS were still shielding and were eligible for free rapid antigen tests. All participants were encouraged to report infection episodes either to the research team or their NHS contact. We collated all available data on COVID-19 infections in the COVMS cohort from their records and performed further testing for seroconversion to anti-N IgG in all samples. Fifty-five (24%) COVMS participants self-reported at least one COVID-19 infection between 28 January 2020 and 25 April 2022. At 12 months post-third dose, the cumulative incidence of COVID-19 infection was 0.23 (95% CI 0.15 to 0.31) in pwMS on BCDT and 0.20 (95% CI 0.11 to 0.28) in those not on BCDT (p=0.6, figure 4). Twenty-two (9.6%) pwMS attended a CMDU at least once, with 17/22 (77.3%) on BCDT. Treatments received were: nirmatrelvir-ritonavir (10/22 (45.5%)), molnupiravir (2/22 (9.0%)), sotrovimab (6/22 (27.3%)) and 4/22 (18.2%) were ineligible for treatment. No hospitalisations or deaths due to COVID-19 occurred.

### Neutralisation of seasonal coronaviruses and SARS-CoV-2 in pwMS

A unique feature of our cohort is that sampling occurred either before or extremely early in the pandemic. We found a subset of our cohort of pwMS had detectable nAbTs against certain SARS-CoV-2 variants in early 2020 (figure 1). Thirty-eight (16.7%) pwMS were sampled before any SARS-CoV-2 antigen exposure (anti-S IgG negative and anti-N IgG negative, online supplemental figure 1B), and yet remarkably we found detectable nAbTs in serum against Ancestral (36/38; 95%), BA.1 (20/38; 53%) and BA.2 (32/38; 84%) regardless of BCDT (online supplemental figure 1A). All (20/20; 100%) of the samples containing nAbTs against BA.1, and 31/32 (97%) against BA.2, also had nAbTs against the Ancestral variant (online supplemental figure 1B–D). All (38/38; 100%) samples had detectable nAbTs (>40) to HCoV-OC43, before exposure (online supplemental figure 1E–G).

### Association between duration of B-cell depleting therapy and vaccine responses

As B-cell depletion resulted in overall lower nAbT, we hypothesised that longer duration of BCDT would associate with reduced nAbT. We used a regression analysis (adjusted for age, sex, anti-N IgG and time since third dose), finding increased BCDT duration significantly reduced nAbTs against all variants except Omicron BA.2 (p=0.19 against Omicron BA.2, p<0.05 across all other variants, online supplemental figure 2). Each additional week of BCDT decreased nAbTs against Omicron BA.5 by 4% (9% CI 1% to 6%, p=0.002).

## Discussion

While COVID-19 has become a mild illness for the majority adult population, pwMS continue to be considered at high risk for severe disease and are offered additional vaccines. While the OCTAVE-DUO^22^ randomised controlled trial (RCT) found overall attenuated vaccine responses across a wide range/heterogenous population of immunocompromised adults, there are no data from RCTs focusing specifically on pwMS. In our prospective observational study, we found that most pwMS generated nAbTs comparable to HVAs post-third vaccine. While pwMS on BCDT generated lower nAbTs following prime-boost vaccination, we did not find an association between lower nAbT and COVID-19 hospitalisation risk. Furthermore, we found that sera pre-COVID-19 from some pwMS contained broadly cross-reactive antibodies to both seasonal and SARS-CoV-2 variants.

Mortality from COVID-19 fell dramatically after initial vaccine roll-out in 2021, including pwMS.^23^ No definitive ‘correlate of protection’ (CoP), induced by COVID-19 vaccines, to use as a proxy for protection has been defined. NAbs against specific variants associate best with protection in most studies, but are only available as a research tool and are estimated retrospectively.^24^ Reassuringly, the post-third dose nAbT in pwMS in our study exceeded the defined CoP against alpha and delta.^25 26^ Few other studies have tested nAbTs in pwMS—largely limited to the first two doses of both mRNA and adenovirus-vectored COVID-19 vaccines.^27 28^ Five studies reported reduced nAbTs after a third COVID-19 vaccine dose in pwMS taking versus not taking BCDT. These studies were limited by the number of variants tested (only Ancestral and Omicron BA.1),^29^ small cohort sizes^30^ or use of a pseudovirus as opposed to live virus microneutralisation assay to determine nAbT. Inconsistencies in Spike expression on pseudoviruses can result in variable nAbT estimates, particularly at lower titres, a significant concern in people on BCDT who have attenuated antibody production.^31^ Only one study described a comparable nAbT response post-third dose between pwMS not on BCDT and HVA (n=70). However, this was also limited to anti-Ancestral nAbTs and reported reduced postvaccine anti-S IgG and nAbTs solely in ocrelizumab-treated pwMS.^31^ In contrast, we found breadth of nAbTs after the third vaccine dose in a large cohort of participants stratified by DMT, with direct comparison with matched HVAs. Our data thus add considerably to the literature by showing crucial differences in the vaccine response in pwMS between those on BCDT and those on other DMTs.

We demonstrated that, as expected, longer duration of BCDT was associated with lower nAbTs to all variants. This finding contrasted with a study of 130 participants, which reported no association between BCDT with anti-CD20 treatment duration and nAbTs postdose three.^32^ Their cohort was predominantly treated with rituximab (76.9%), compared with ocrelizumab in our cohort, and participants were older (median age 48 vs 39 in COVMS). Our data both support JCVI recommendations to extend additional COVID-19 vaccine doses to pwMS, and guidance from the National Multiple Sclerosis Society, who advise vaccinating 2–4 weeks before starting anti-CD20 therapy.^14^ Treatment breaks for vaccination should be informed by the specific DMT, using mounting evidence for time to B-cell repopulation from cessation of each DMT, and data on the effect of delaying medication on MS.^33^

Current NHS guidance on vaccination of pwMS applies regardless of DMT. More research is needed on nAbT waning in this cohort and to determine whether ongoing COVID-19 booster vaccines are needed for pwMS on non-DT. PwMS were advised to shield between the beginning of lockdown and Autumn 2021, which was associated with anxiety and other mental health problems.^34^ Data on hospitalisation risk post-third dose in pwMS are limited to a number of small studies describing hospitalisation rates of <2% (including those on BCDT) up to late 2022.^35^ There are no data on hospitalisation rates in pwMS after this date, and thus after the Omicron waves.^36^ Since 2022, CMDUs have provided early COVID-19 treatments for higher-risk patients, to try and avoid severe sequelae through early antiviral drugs. Interestingly, 33/55 (60%) of our participants with a COVID-19 infection did not access a CMDU, suggesting a mild infection, and that vaccination alone conferred protection against severe disease in those patients. We were not able to disaggregate if anti-SARS-CoV-2 monoclonal antibody therapy, given as a COVID-19 treatment, impacted on nAbT. Our data, with follow-up to 2024, justifies a cautious de-escalation of the ‘vulnerable’ status of pwMS, particularly those not on BCDT, but is dependent on ongoing surveillance and rapid risk assessment of emerging, potentially immune-evasive variants.

NAb responses to vaccines in anti-CD20-treated patients for non-MS conditions have been generalised to pwMS without quality prospective data. In haematological malignancies, for example, anti-CD20 therapy is administered over a shorter course; at higher doses or as a different monoclonal antibody.^37^ Neutralising immunity after COVID-19 vaccinations is also affected by the underlying disease. As expected, therefore, the effect of anti-CD20 therapy on SARS-CoV-2 nAbTs in our cohort was markedly different to the cohort of people with haematological malignancies described by the CAPTURE (COVID-19 antiviral response in a pan-tumour immune monitoring) study.^37^ They reported no detectable nAb response post-third vaccine in anti-CD20-treated patients, while 11/28 (39.3%) of anti-CD20-treated patients in our study had detectable (IC_50_ >40) antibodies. The OCTAVE-DUO (Ongoing COVID-19 Trial of Additional Vaccine Efficacy) study^22^ investigated nAb responses to COVID-19 vaccines in immunocompromised patients (excluding MS) and found BCDT significantly increased the risk of serological non-response to COVID-19 vaccine. Only 6/44 (13.6%) non-responders post-third vaccine had received BCDT. This highlights the need for more research into other determinants of vaccine response, particularly to disaggregate effects of BCDT from disease indication, comorbidities, prior or concurrent treatment exposures and patient demographic factors.

We observed an intriguing ability to neutralise SARS-CoV-2 variants in pwMS. Sera from early 2020 had high nAbT in pwMS directed against HCoV-OC43, a common coronavirus, which predates COVID-19.^38^ These samples were taken before COVID-19 vaccination, and before the existence of Omicron BA.1 or BA.2, in participants with no prior exposures recorded and with a negative anti-N IgG. These same sera neutralised Ancestral SARS-CoV-2 and, surprisingly, Omicron BA.1 and BA.2, nearly 2 years before it evolved. This response was maintained in pre-third dose samples, which were taken between 8 September and 13 October 2021, again before the first recorded Omicron case in the UK (27 November 2021).^39^ To our knowledge, no other group has reported the presence of sera, which appears to display pre-existing, pan-coronavirus neutralising activity against a wide suite of SARS-CoV-2 variants and seasonal HCoV-OC43 in pwMS. Molecular mimicry and cross-reactivity, between antibodies against Epstein-Barr virus and glial cell adhesion protein in the central nervous system, is thought to contribute to MS pathogenesis.^40^ Similar antibody perturbations might also influence coronavirus immunity, potentially providing protection against severe COVID-19. This intriguing hypothesis requires further investigation, including analyses of individual spike-specific memory B cells and their corresponding antibody.

## Limitations

While our study is the largest and most comprehensive study of vaccine responses in pwMS, it has limitations. We were not able to evaluate DMTs separately, due to limited cohort size and dosing schedules, and thus could not evaluate the effect of specific DMTs on nAbTs. Some infection episodes may have been missed, due to failure to seroconvert to anti-N antibodies in the context of B-cell depletion, incomplete participant reporting of infection or asymptomatic infections. Every attempt was made to collect these, and this was an engaged cohort with access to free testing. Risk-avoidance behaviour data (eg, shielding) was not collected. In the comparison of pre-third and post-third vaccination nAbTs, most individuals were unpaired (n=188), with only a small subset having paired sera (n=18). Consequently, unpaired statistical tests were applied, which may not fully account for within-individual variation.

## Conclusion

PwMS on non-DT had a comparable vaccine response to a healthy matched comparator cohort. Longer BCDT duration significantly reduced nAbTs post-third dose but did not increase infection risk. PwMS exhibit broad cross-reactivity to seasonal coronaviruses and some SARS-CoV-2 variants, potentially explaining low severe COVID-19 incidence in this cohort. Individualised vaccination strategies for pwMS could be stratified based on DMT.

## Supplementary material

10.1136/bmjno-2025-001210 online supplemental file 1

10.1136/bmjno-2025-001210 online supplemental file 2

10.1136/bmjno-2025-001210 online supplemental file 3

## Data Availability

Data are available on reasonable request.
