# Supplementary material for: Immunogenicity of three-dose COVID-19 vaccines in people living with multiple sclerosis
Source: BMJ Neurol Open. 2025 Dec 16;7(2):e001210. doi: 10.1136/bmjno-2025-001210 (PMC12716506; doi:10.1136/bmjno-2025-001210)
Supplement: online supplemental file 1 [file bmjno-7-2-s001.pdf]

Supplemental Figure 1

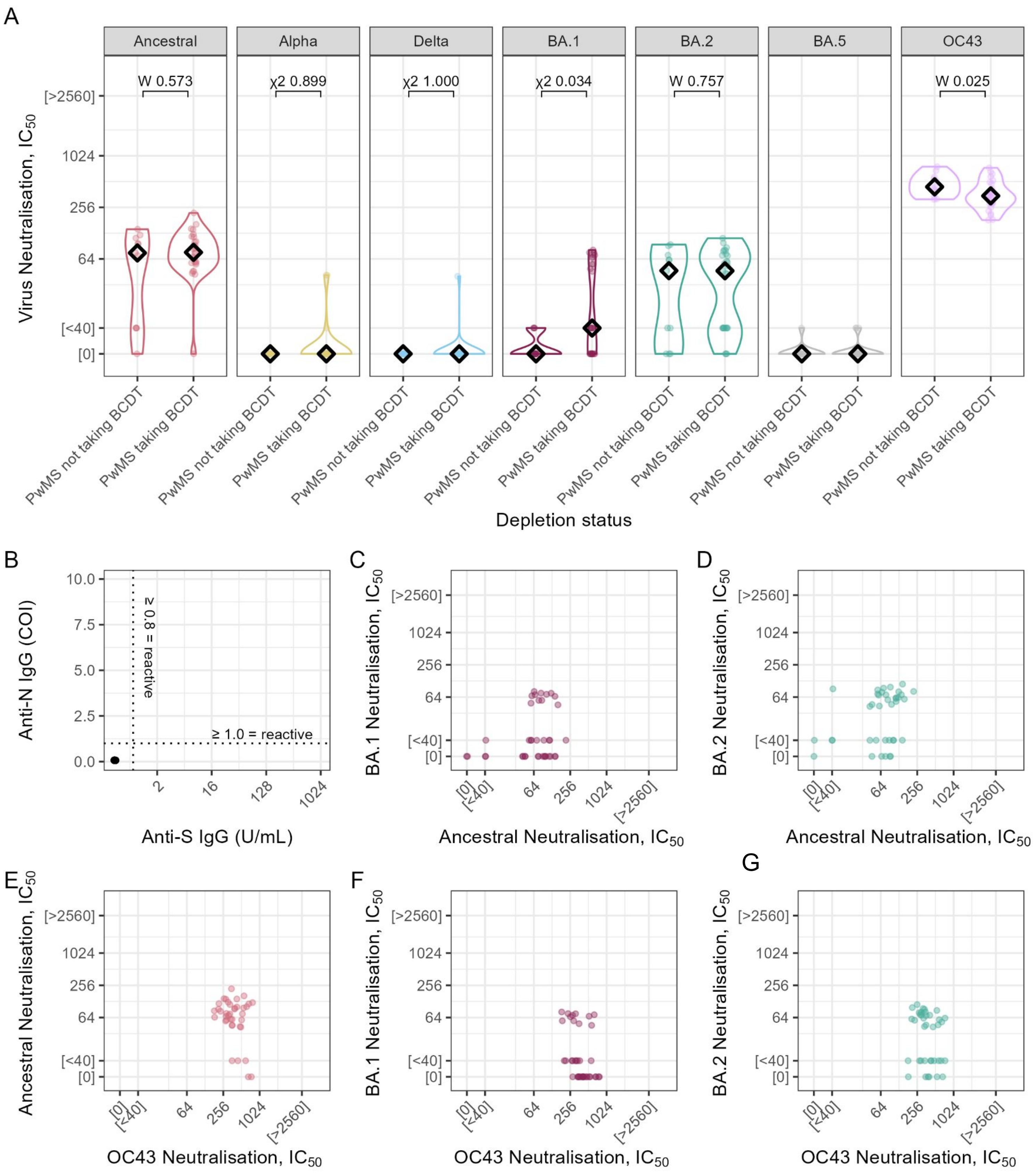

Supplemental Figure 1. Neutralising antibodies against Ancestral, BA.1 and BA.2 variants were observed prior to evidence of antigen exposure

Serum neutralisation titres (IC_{50}) against SARS-CoV-2 variants and HCoV-OC43 in samples collected prior to SARS-CoV-2 antigen exposure (pre-vaccination, pre-infection and non-reactive to anti-N IgG (cutoff index <1)) (A) IC_{50} pre-antigen exposure for pwMS receiving (BCDT) and not receiving (non-DT) B-cell depleting therapy. (B) Anti-S IgG levels compared to Anti-N IgG levels. (C) IC_{50} pre-antigen exposure against Ancestral compared to BA.1 variant. (D) IC_{50} pre-antigen exposure against Ancestral compared to BA.2 variant. (E) IC_{50} pre-antigen exposure against Ancestral variant compared to OC43 (F) IC_{50} pre-antigen exposure against OC43 compared to BA.1 variant. (G) IC_{50} pre-antigen exposure against OC43 compared to BA.2 variant.
