# Supplementary material for: Immunogenicity of three-dose COVID-19 vaccines in people living with multiple sclerosis
Source: BMJ Neurol Open. 2025 Dec 16;7(2):e001210. doi: 10.1136/bmjno-2025-001210 (PMC12716506; doi:10.1136/bmjno-2025-001210)
Supplement: online supplemental file 2 [file bmjno-7-2-s002.pdf]

Supplemental Figure 2

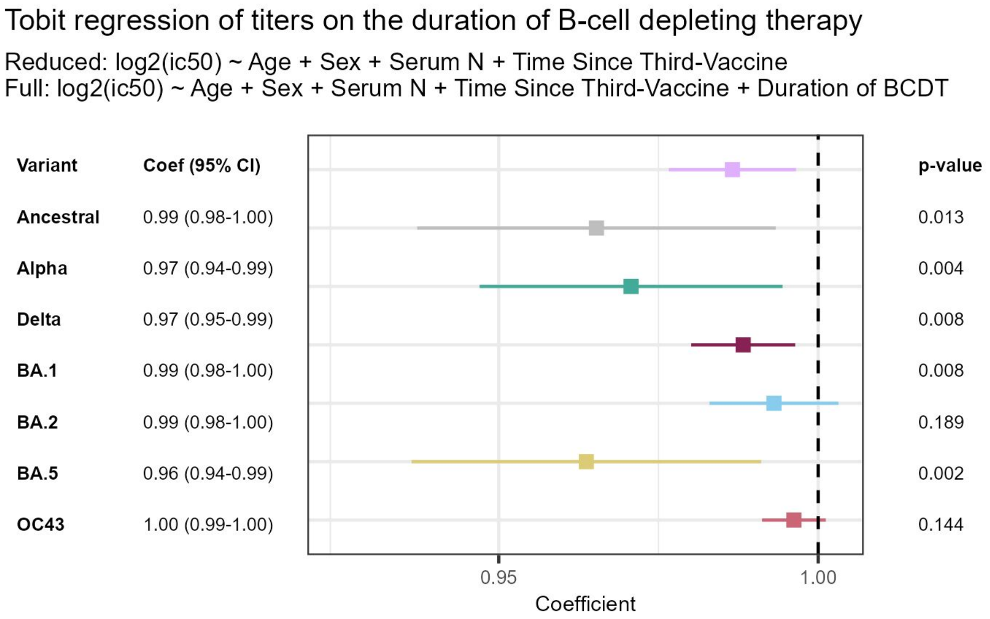

Supplemental Figure 2. An increased duration of B-cell depleting therapy course was associated with reduced neutralising antibody titres against Ancestral, Alpha, Delta, BA.1 and BA.5 variants Tobit regression of serum neutralisation titres (IC_{50}) against SARS-CoV-2 variants and HCoV-OC43 on participant characteristics and duration of B-cell–depleting therapy. Samples were collected from people with MS taking B-cell depleting after a third-vaccine. Multivariable Tobit regression models were fitted separately for each variant with IC_{50} as the dependant variable. Statistical tests were conducted with likelihood-ratio tests (p-value) comparing the full modell (Full) and a a nested reduced model (Reduced), with and without the term for duration of B-cell–depleting therapy, measured in weeks.
