# Supplementary material for: Immunogenicity of three-dose COVID-19 vaccines in people living with multiple sclerosis
Source: BMJ Neurol Open. 2025 Dec 16;7(2):e001210. doi: 10.1136/bmjno-2025-001210 (PMC12716506; doi:10.1136/bmjno-2025-001210)
Supplement: online supplemental file 3 [file bmjno-7-2-s003.docx]

**Supplemental Table 1**

|  | **Legacy** | | |
| --- | --- | --- | --- |
| **Characteristic** | **Paired samples taken pre and post-third vaccine**, N = 38*^1^* | **Samples taken Pre-third vaccine only**, N = 47*^1^* | **Samples taken Post-third vaccine only**, N = 4*^1^* |
| Sex |  |  |  |
| Female | 25 (66%) | 36 (77%) | 2 (50%) |
| Male | 13 (34%) | 11 (23%) | 2 (50%) |
| Median age (years) [IQR] | 48 [35-56] | 39 [33-47] | 44 [35-56] |
| First dose |  |  |  |
| AZD1222 | 5 (13%) | 13 (28%) | 1 (25%) |
| BNT162b2 | 31 (82%) | 33 (70%) | 3 (75%) |
| mRNA1273 | 0 (0%) | 1 (2.1%) | 0 (0%) |
| others | 2 (5.3%) | 0 (0%) | 0 (0%) |
| Second dose |  |  |  |
| AZD1222 | 5 (13%) | 13 (28%) | 1 (25%) |
| BNT162b2 | 31 (82%) | 33 (70%) | 3 (75%) |
| mRNA1273 | 0 (0%) | 1 (2.1%) | 0 (0%) |
| others | 2 (5.3%) | 0 (0%) | 0 (0%) |
| Third dose |  |  |  |
| BNT162b2 | 38 (100%) | 47 (100%) | 4 (100%) |
| *^1^*n (%); Median [25%-75%] | | | |

**Author contributions:**

Investigation: M.S.-T., D.G., A.H., D.C., S.S., A.A., A.B., S.A.-A., Y.H., C.Y., S.M., R.C., M.M., A.Ho., G.D., R.P., T.S., P.S.-L., J.B., R.H., S.S.-L., [A.Ao](http://a.ao)., E.J.C., M.Y.W., O.C., E.C.W. Methodology: M.S.-T., D.G., A.H., M.Y.W., E.J.C., D.L.V.B., E.C.W, O.C., Formal Analysis: M.S.-T., D.G., E.J.C., D.L.V.B., Visualisation: M.S.-T., D.G., E.J.C., Data Curation: M.S.-T., D.G., A.H., A.Ho., G.D., R.P., T.S., P.S.-L., J.B., R.H., D.C., S.S., A.A., A.B., S.A.-A., Y.H., C.Y., S.M., R.C., M.M., Writing – original draft: M.S.-T., D.G., E.J.C., E.C.W., Writing – review & editing: M.S.-T., D.G., A.H., A.Ho., G.D., R.P., T.S., P.S.-L., J.B., R.H., D.C., S.S., A.A., A.B., S.A.-A., Y.H., C.Y., S.M., R.C., M.M., V.L., G.K., S.Ga., N.S.L., B.W., C.S., S.Ga., D.L.V.B., E.J.C., E.C.W., O.C., Project Administration: M.S.-T., E.C.W., O.C., Resources: V.L., G.K., S.Ga., N.S.L., B.W., C.S., [S.Ga](http://s.ga)., M.Y.W., Supervision: V.L., G.K., S.Ga., N.S.L., B.W., C.S., S.Ga., M.Y.W., E.J.C., D.L.V.B., E.C.W., O.C., Funding Acquisition: E.C.W., O.C., Conceptualisation: M.S.-T., D.G., A.H., E.C.W., O.C., M.Y.W., D.L.V.B., E.J.C., Validation: G.K., D.L.V.B., E.J.C., Decision to Submit the Manuscript: M.S.-T., A.H., D.G., E.J.C., D.L.V.B., E.C.W., O.C.

**All authors reviewed and approved the final manuscript.**

**M.S.-T., D.G. and A.H. all contributed equally to the work.**

**E.C.W., O.C., M.W. and E.J.C. all contributed equally to the work.**

**E.C.W. is the guarantor.**

**No competing interests to declare for any authors.**

**Consortium authors:**

**Crick COVID Serology Pipeline:**  Ashley S Fowler, Murad Miah, Callie Smith, Mauro

Miranda, Philip Bawumia, Harriet V Mears, Lorin Adams, Emine Hatipoglu, Nicola O’Reilly,

Scott Warchal, Karen Ambrose, Amy Strange, Gavin Kelly, Svend Kjaer

**Legacy Investigators:**Rupert CL. Beale, Padmasayee Papineni, Tumena Corrah, Richard

Gilson

**Acknowledgements:**

The authors would like to thank all the study participants, the staff of the NIHR Clinical Research Facility at UCLH including Miguel Alvarez and Marivic Ricamara. We would like to thank the staff of the Scientific Technology Platforms (STPs) and COVID-19 testing pipeline at the Francis Crick Institute. We thank Prof. Alex Sigal, Dr Khadija Khan and Prof. Tulio de Oliveira of Africa Health Research Institute, Durban, South Africa, Dr Laura McCoy of UCL, and Prof. Gavin Screaton of the University of Oxford, for providing source material.

**Funding**

This study was funded by the NIHR (National Institute for Health and Care Research) UCLH BRC (Biomedical Research Centre), The Multiple Sclerosis Society (Code: 92), and the NIHR Research Professorship to O. Ciccarelli (RP-2017-08-ST2-004). The views expressed are those of the authors and not necessarily those of the NIHR or the Department of Health and Social Care. The study was also supported by the UK MS Society (Grant Code 92).

This research was funded in whole, or in part, by the Wellcome Trust [CC2166, CC1283, CC1114, CC2230, CC2060, CC2041, CC0102). This work was supported by the National Institute for Health Research University College London Hospitals Department of Health’s NIHR Biomedical Research Centre (BRC, RP-2017-08-ST2-004), ECW, VL and BW are supported in whole or in part by the BRC. Further funding by the UK Research and Innovation and the UK Medical Research Council (MR/W005611/1, MR/Y004205/1, and MR/X006751/1 to EJC), and by the Francis Crick Institute which receives its core funding from Cancer Research UK (CC2166, CC1283, CC1114, CC2230, CC2060, CC2041, CC0102), the UK Medical Research Council (CC2166, CC1283, CC1114, CC2230, CC2060, CC2041, CC0102), and the Wellcome Trust (CC2166, CC1283, CC1114, CC2230, CC2060, CC2041, CC0102). The funders had no role in the preparation of this report or the decision to publish.

**SARS-CoV-2 viral neutralisation assay**

Vero E6 cells (Institute Pasteur), or those expressing ACE2 and TMPRSS2 (VAT-1) (Centre for Virus Research), were cultured to 90-100% confluency in 384-well plates. These cells were initially exposed to different MOIs of each SARS-CoV-2 variant and varying concentrations of a control monoclonal nanobody. This process aimed to standardise for potential variations in replication among variants and identify conditions comparable between variants. Following the calibration process, we exposed cells to serial dilutions of patient serum samples pre-infection. 24 hours after infection, we fixed the cells using a 4% final concentration of Formaldehyde, permeabilized with 0.2% TritonX-100, 3% BSA in PBS (v/v), and stained for SARS-CoV-2 N protein using in-house-produced Biotin-labelled CR3009 antibody in conjunction with Alexa488-streptavidin (ThermoFisher) and cellular DNA using DAPI. We conducted whole-well imaging at 5x magnification using an Opera Phenix (Perkin Elmer), and analysed fluorescence areas and intensity using the Harmony software associated with Phenix (Perkin Elmer). We determined the inhibition levels from the ratio of the measured area of infected cells to the total area occupied by all cells. We assessed the inhibitory profile of each serum sample by fitting a 4-parameter dose-response curve implemented in SciPy. nAbTs are expressed as the fold-dilution of serum necessary to inhibit 50% of viral replication (IC_50_). These titers are further categorised if they exceed the quantitative range (complete inhibition), fall below the quantitative range but still within the qualitative range (indicating partial inhibition), or show no inhibition at all. Thus, values are both left and right censored.
